# Transcriptome profiling using pyrosequencing shows genes associated with bast fiber development in ramie (*Boehmeria nivea* L.)

**DOI:** 10.1186/1471-2164-15-919

**Published:** 2014-10-22

**Authors:** Jie Chen, Zhihua Pei, Lunjin Dai, Bo Wang, Lijun Liu, Xia An, Dingxiang Peng

**Affiliations:** MOA Key Laboratory of Crop Ecophysiology and Farming System in the Middle Reaches of the Yangtze River, College of Plant Science and Technology, Huazhong Agricultural University, No. 1 Shizishan Street, Hongshan District, Wuhan, 430070 Hubei Province China; National Key Laboratory of Crop Genetic Improvement, Center for Bioinformatics, College of Life Science and Technology, Huazhong Agricultural University, Hongshan District, Wuhan, 430070 Hubei Province China

**Keywords:** Ramie, Bast fiber, Transcriptome, Cellulose synthase, Expansin, Xyloglucan endotransglucosylase/hydrolase

## Abstract

**Background:**

Ramie (*Boehmeria nivea* L.), popularly known as “China grass”, is one of the oldest crops in China and the second most important fiber crop in terms of area sown. Ramie fiber, extracted from the plant bast, is important in the textile industry. However, the molecular mechanism of ramie fiber development remains unknown.

**Results:**

A whole sequencing run was performed on the 454 GS FLX + platform using four separately pooled parts of ramie bast. This generated 1,030,057 reads with an average length of 457 bp. Among the 58,369 unigenes (13,386 contigs and 44,983 isotigs) that were generated through *de novo* assembly, 780 were differentially expressed. As a result, 13 genes that belong to the cellulose synthase gene family (four), the expansin gene family (three) and the xyloglucan endotransglucosylase/hydrolase (XTH) gene family (six) were up-regulated in the top part of the bast, which was in contrast to the other three parts. The identification of these 13 concurrently up-regulated unigenes indicated that the early stage (represented by the top part of the bast) might be important for the molecular regulation of ramie fiber development. Further analysis indicated that four of the 13 unigenes from the expansin (two) and XTH (two) families shared a coincident expression pattern during the whole growth season, which implied they were more relevant to ramie fiber development (fiber quality, etc.) during the different seasons than the other genes.

**Conclusions:**

To the best of our knowledge, this study is the first to characterize ramie fiber development at different developmental stages. The identified transcripts will improve our understanding of the molecular mechanisms involved in ramie fiber development. Moreover, the identified differentially expressed genes will accelerate molecular research on ramie fiber growth and the breeding of ramie with better fiber yields and quality.

**Electronic supplementary material:**

The online version of this article (doi:10.1186/1471-2164-15-919) contains supplementary material, which is available to authorized users.

## Background

Ramie (*Boehmeria nivea* L.), usually called “China grass”, is a perennial herbaceous plant in the nettle family, *Urticaceae*. Ramie fiber, generated from the stem bast, is an important natural fiber in the textile industry because of the ability to retain shape, reduce wrinkling and introduce a silky luster to the appearance of a fabric [1]. Nevertheless, the flaws, such as resistance to dyeing, confined elasticity and elongation potential and the stiff and brittle nature of the cloth, have encouraged breeding of ramie with better fiber quality. Thus, focusing on the developmental process will benefit in improving ramie fiber yield and quality. To increase our understanding of ramie fiber development and related molecular mechanism, researchers have cloned genes that may related, including *GalAT*[2], *Actin*[3], *CesA*[4], *COMT*[5] and *UDPGDH*[6]. However, the restricted single-gene cloning strategy has limited benefits. One way to improve the process is the application of the next-generation sequencing platform, including the 454 FLX Instrument, the ABI SOLiD System and the Illumina Genome Analyzer, which have been applied very successfully in previous studies [7–13]. For example, the ramie universal transcriptome has been determined, with a preliminary quantification of 51 *CesA* (cellulose synthase) genes [11]. However, there has been no further research related to ramie fiber development, especially at different growth stages.

A previous study on ramie was carried out using the cultivar, “ZhongZhu 1” [11], which is an elite variety with good fiber quality (with a fiber fineness of about 2000 m/g). However, we chose “1504” (with a fiber fineness of about 2800 m/g) as the best qualified material to study ramie fiber development. In field production, the growth period of ramie fiber could be roughly divided into initiation, fast growth and maturation stages. However, these descriptions did not meet the need for exact experimental conditions, and the timings of their boundaries are vague, which vary in the different growth seasons of ramie. Previous studies on ramie used a fixed timeline [11, 14], which may be appropriate for other experiments (e.g., for drought stress), but was not suitable for studies of ramie fiber development. Fortunately, previous studies had extracted the fiber of flax from stem bark [15, 16], which we then followed as the sampling method.

Plant cell walls are complex structures composed of polysaccharides, proteins, and lignins. Among the cell wall polysaccharides, cellulose is the main load-bearing wall component [17]. It is the first and most important family in the cell wall, especially with regards to fiber growth and development. Genes in the cellulose synthase superfamily have been studied in model plants, such as rice [18] and *Arabidopsis*[17]. Expansin, known to have cell wall loosening activity and is involved in cell expansion and other developmental events during which cell wall modification occurs [19], plays important roles during cell elongation [20] and expansion [21], and specifically regulates fiber elongation [22] and fruit ripening [23]. Other studies have highlighted the potential relationship between expansin genes and fiber growth in cotton [24, 25]. Moreover, the polysaccharide, xyloglucan, is thought to play an important structural role in the primary cell wall of dicotyledons [26]. Consequently, the xyloglucan endotransglucosylase/hydrolase (XTH) family that was shown to be involved in the formation of the secondary cell walls of vascular tissue [27] (especially in regulating cotton fiber elongation [28–30]) indicating the importance of this gene family in studying ramie fiber development. In this study, the ramie transcriptome was constructed from mixed samples, and the differentially expressed genes among these samples were used to verify potential genes that may be related to ramie fiber development. We inferred that the early developmental stage (represented by the top part of the stem bark sample) of ramie fiber growth may play a more important role in the whole developmental period, and the 13 genes that all-up-regulated (respectively up-regulated in one of the four samples than rest three) in sample T (top part of the bast), especially the four from the expansin (two) and XTH (two) gene families, should be subjected to further in-depth characterization.

## Results

### *De novo*assembly of the transcriptome library

The read lengths of four separately pooled samples, which were evenly distributed throughout the ramie stem (stem shoot with Leaves: L; Top part of stem bark: T; Middle part of stem bark: M; Bottom part of stem bark: B), as well as the overall assembled sequences, are shown in Table 1 and Additional file 1. The average read lengths were 449, 453, 442 and 460 bp for the four samples, respectively. The whole run generated 1,030,057 reads with an average length of 457 bp. After removing the low-quality reads (Q value <20), the clean reads were assembled *de novo* by the Newbler software, which is frequently used in *de novo* pyrosequencing projects [31], using default settings. As a result, 17,881 contigs and 52,996 isotigs were generated. After elimination of redundancy, 58,369 unigenes (13,386 contigs and 44,983 isotigs) were generated; the contigs had an average length of 1,343 bp (Table 1). Among the unigenes, 51,025 (87.42%) and 8,025 (13.75%) sequences were longer than 200 bp and 1000 bp, respectively. The lengths of the assembled unigenes ranged from 90 to 7641 bp (Additional file 1).

**Table 1:** **Distribution lengths of the separately pooled samples and the overall data**

Samples	L	T	M	B	All	Contigs
Reads	276423	286948	254985	211701	1030057	-
Average lengths (bp)	449	453	442	460	457	1343

### Gene annotation and functional pathway construction

The GetORF tool from EMBOSS [32] was used for gene prediction, which identified all of the assembled contigs and isotigs to encode protein sequences and were qualified for further annotation. The predicted protein sequences were then annotated to the non-redundant protein databases in GenBank and Swiss-Prot using BLASTp under the threshold of 1e-5. The first entry was regarded as the annotation information for the corresponding unigenes. However, there might be a situation where the “true” BLAST result was concealed by the first non-informative entry (e.g., an unnamed protein product) when choosing the first entry as the annotation information. To overcome this obstacle, we repeated the BLAST of the unigenes that had been annotated to meaningless results, and the output results were expanded to 15 entries (if possible) for manual selection. As a result, 156,970 entries from 11,555 unigenes (2645 contigs and 8910 isotigs) were obtained (Additional file 2) for further selection. Finally, we altered the annotation information of 6677 unigenes (1780 contigs and 4897 isotigs, Additional file 3), which resulted in the annotation of 7065 from 13,386 contigs and 18,006 from 44,983 isotigs (Additional file 4). Meanwhile, the predicted protein sequences were compared with the Swiss-Prot and TrEMBL databases using BLASTp (under the threshold of 1e-5). The 11,330 (19.41%) retrieved protein sequences were then matched to 6283 Gene Ontology (GO) terms (Additional file 5, lists detailed in Additional file 6) using the GoPipe software [33]. In addition, the predicted protein sequences were compared to the Kyoto Encyclopedia of Genes and Genomes (KEGG) database [34] using a bidirectional BLAST under the threshold of 1e-5, which resulted in a total of 3076 KO (KEGG Orthology) numbers that corresponded to particular sequences. The pathway distribution is shown in Additional file 7 and detailed in Additional file 8.

### Differential expression redundancy and enrichment analyses

**Figure 1:**
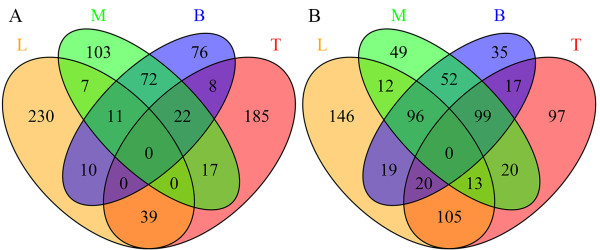
**Numbers of unigenes that were differentially expressed in the four samples.** Numbers displayed were either up-regulated **(A)** or down-regulated **(B)** in the four samples, which were stem shoot with leaves (sample L), top part of stem bark (sample T), middle part of stem bark (sample M) and bottom part of stem bark (sample B). Numbers of unigenes that shared simultaneously differentially expressed patterns in more than one sample displayed at the intersections of the correspondent samples.

As indicated in Table 2, hundreds of unigenes had differential expression levels between each combination of two samples, except for the comparison of samples M and B (only 28 unigenes were up-regulated and 16 were down-regulated). The numbers of differentially expressed unigenes in each sample are displayed in Figure 1, which shows that there were 297, 271, 232 and 199 unigenes up-regulated in samples L, T, M and B, respectively; while 411, 371, 341 and 338 unigenes were down-regulated, respectively. Altogether 780 unigenes were differentially expressed (listed in Additional file 9 and detailed in Additional file 10). Notably, the numbers of up- and down-regulated unigenes in the sample combination M and B (105 up and 247 down, Figure 1) were greater than in the other two-sample combinations (up/down: L and T, 39/133; L and M, 18/121; L and B, 21/135; T and M, 39/132; T and B, 30/136; M and B, 105/247, detailed in Figure 1), which coincided with the lowest numbers of differentially expressed unigenes between M and B (28 up and 16 down, Table 2). This suggested that either the degree of fiber development was similar between samples M and B or gene regulation only has a minor influence on fiber growth during the later developmental stages.

**Table 2:** **Numbers of unigenes that had differential expression patterns between each two-sample combination**

Samples	Up-regulated	Down-regulated
L/T	196	196
L/M	199	191
L/B	173	151
T/M	161	134
T/B	165	125
M/B	28	16

Numbers were calculated by comparing the earlier samples to the latter ones. For instance, numbers in the last row represented 28 and 16 unigenes were up- and down-regulated in sample M compared to sample B. Unigenes were up-regulated when the q-value < 0.001 and log_2_(Fold_change) normalized >1, or down-regulated when the q-value < 0.001 and log_2_(Fold_change) normalized < -1 (detailed in Additional file 10).

Among the differentially expressed unigenes, the enrichment of GO terms (Table 3) and KEGG pathways (Table 4) was dependent on the ratio of divergence to all unigenes (false discovery rate (FDR) <0.01). As indicated in Table 3, the two conjunct GO terms enriched in sample T (compared with L, M and B) were the external encapsulating structure (GO: 0030312) and the extracellular region (GO:0005576). Thus, the enrichment of conjunct GO terms in one of the four samples compared with the other three happened only in sample T, which implied that the early stage of ramie fiber development (represented by sample T) may be more sensitive to molecular regulation. Meanwhile, the numbers of unigenes that made up these two GO terms were fairly small (388 for the external encapsulating structure and 681 for the extracellular region, shown in Additional file 5 and listed in Additional file 6). Additionally, four KEGG pathways: pathogenic *Escherichia coli* infection; the gap junction; the photosynthesis-antenna proteins and the cutin and the suberine and the wax biosynthesis pathways (Table 4) were simultaneously enriched in sample L compared with the other three samples.

**Table 3:** **GO terms that were enriched in each sample combination**

Samples	GO-ID	GO terms	GO classes	P value	FDR
L/T	GO:0030312	External encapsulating structure	component	2.72E-11	1.55E-09
	GO:0005198	Structural molecule activity	function	1.09E-10	2.06E-09
	GO:0005576	Extracellular region	component	9.34E-11	2.06E-09
	GO:0016209	Antioxidant activity	function	0.000263	0.003746
	GO:0008152	Metabolism	process	0.000998	0.008127
	GO:0009058	Biosynthesis	process	0.000778	0.008127
	GO:0009986	Cell surface	component	0.00098	0.008127
	GO:0016829	Lyase activity	function	0.001216	0.008667
L/M	GO:0016491	Oxidoreductase activity	function	2.85E-05	0.001622
	GO:0005198	Structural molecule activity	function	0.000121	0.003445
L/B	GO:0005576	Extracellular region	component	2.35E-05	0.000669
	GO:0016491	Oxidoreductase activity	function	1.87E-05	0.000669
T/M	GO:0030312	External encapsulating structure	component	3.52E-14	2.01E-12
	GO:0005576	Extracellular region	component	3.34E-12	9.52E-11
	GO:0043170	Macromolecule metabolism	process	0.000327	0.006221
T/B	GO:0005576	Extracellular region	component	1.63E-11	9.28E-10
	GO:0030312	External encapsulating structure	component	7.05E-10	2.01E-08
	GO:0005198	Structural molecule activity	function	9.32E-07	1.77E-05

**Table 4:** **KEGG pathways that were differentially expressed between the two-sample combinations**

Samples	KEGG pathways	P value	FDR
L/T	Ribosome	5.55E-09	1.72E-06
	Pathogenic Escherichia coli infection	8.34E-06	0.001297
	Cutin, suberine and wax biosynthesis	1.69E-05	0.001753
	Photosynthesis - antenna proteins	6.01E-05	0.003741
	Gap junction	5.88E-05	0.003741
L/M	Pathogenic Escherichia coli infection	4.18E-09	1.30E-06
	Cutin, suberine and wax biosynthesis	3.74E-07	5.81E-05
	Gap junction	5.77E-07	5.99E-05
	Phagosome	2.15E-05	0.00167
	Glucosinolate biosynthesis	4.96E-05	0.003086
	Photosynthesis - antenna proteins	9.81E-05	0.00436
	Phenylpropanoid biosynthesis	9.56E-05	0.00436
	Protein processing in endoplasmic reticulum	0.000231	0.008971
L/B	Pathogenic Escherichia coli infection	3.69E-09	1.15E-06
	Photosynthesis - antenna proteins	7.03E-07	8.70E-05
	Gap junction	8.39E-07	8.70E-05
	Cutin, suberine and wax biosynthesis	6.44E-06	0.0005
	Legionellosis	1.95E-05	0.001215
	Phagosome	4.25E-05	0.002202
T/M	Phenylpropanoid biosynthesis	1.92E-05	0.005956
	Cysteine and methionine metabolism	6.39E-05	0.009935
M/B	Protein processing in endoplasmic reticulum	1.39E-05	0.00433

### Unigenes up-regulated in the top part of the bast (sample T)

**Figure 2:**
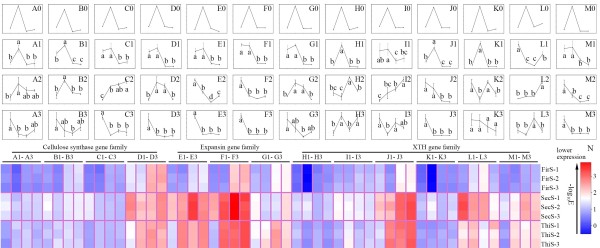
**Relative expression quantities of the 13 unigenes.** The relative expression levels within the four parts (samples L, T, M and B) among the 13 unigenes (columns **A** to **M**, which contained four unigenes (isotig01514_77, isotig06919_78, isotig06943_25 and isotig07154_19, **A** to **D**) from the cellulose synthase superfamily, three unigenes (isotig10345_23, isotig02054_18 and isotig01251_6, **E** to **G**) from the expansin gene family and six unigenes (HRX1MBH01BLWDW_9, isotig00610_25, isotig01663_22, isotig04660_8, isotig09773_10 and contig04902_19, **H** to **M**) from the XTH gene family, respectively) at three seasons (FirS, SecS and ThiS, which displayed in rows 2 to 4, respectively) are shown by line charts (**A1** to **M3**). Each line chart (**A0** to **M3**) was formed as the order of samples L, T, M and B (X-axis) for their quantitative results (Y-axis). Quantitative qRT-PCR was carried out (using the primers listed in Additional file 12) on the four sample parts (L, T, M, B) in the first (FirS), second (SecS) and third (ThiS) seasons with three biological replicates. The expression levels (using *GAPDH* as control) were transformed (-log_10_E) into colors **(N)** in order to assess their overall expression levels. Meanwhile, the significance analyses were performed within each unigene among four samples, using the Holm-Sidak method of Sigmaplot software, under the P-value of 0.05. The RPKM values that were obtained from the transcriptome sequencing results are also shown (row 1, **A0** to **M0**).

Among all the unigenes that had differential expression patterns, most of the unigenes that belong to the cellulose synthase (four out of five), expansin (three out of four), and the XTH (six out of eight, listed in Additional file 9) gene families were included in the all-up pattern from sample T. Specifically, isotig01514_77, isotig06919_78, isotig06943_25 and isotig07154_19 (Figure 2A, B, C and D, respectively) from the cellulose synthase superfamily; isotig10345_23, isotig02054_18 and isotig01251_6 (Figure 2E, F and G, respectively) from the expansin family; and HRX1MBH01BLWDW_9, isotig00610_25, isotig01663_22, isotig04660_8, isotig09773_10 and contig04902_19 (Figure 2H, I, J, K, L and M, respectively) from the XTH family were highlighted for their coincident all-up expression patterns in sample T (up-regulated in sample T than in samples L, M and B, respectively).

**Figure 3:**
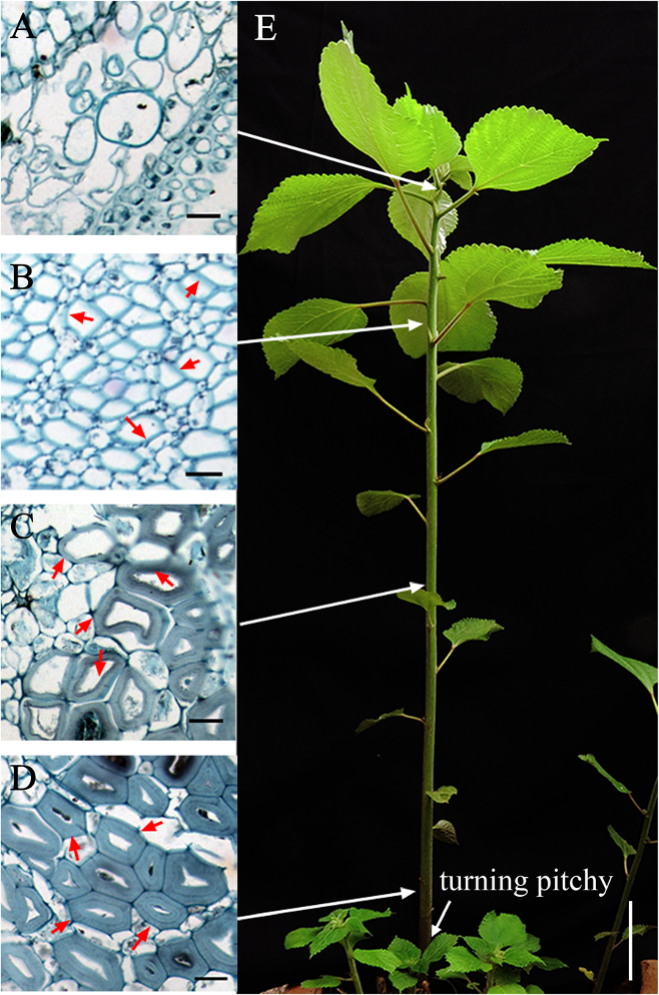
**Brief view of the four samples that represented different fiber developmental stages.** Samples were collected from the start of fiber ripening stage (when the aboveground stem was turning pitchy, as indicated by the arrow in **E**). The slicing results are shown beneath samples L **(A)**, T **(B)**, M **(C)** and B **(D)** and represent their fiber developmental status correspondingly. Arrows pointing from **A**, **B**, **C** and **D** to the ramie stem in **E** show the relative positions of the four samples. The differentially thickened stem fiber cell walls are denoted by arrows (red arrows in **B**, **C** and **D**). Bars in **A**, **B**, **C** and **D** are 20 μm long, whereas in **E** is 10 cm long.

Therefore, we hypothesized that the early fiber developmental stage, represented by sample T (Figure 3), has a more important role in ramie fiber growth than the other stages. Subsequently, these 13 unigenes were picked out for quantitative real-time reverse transcription PCR (qRT-PCR) quantification using similarly pooled samples from the first season (FirS), the second season (SecS) and the third season (ThiS). The overall expression quantities are shown by their colors (Figure 2N) that were translated from the quantification results (-log_10_ E). Generally speaking, the expression pattern trends for FirS (Figure 2A1 to M1) were similar to the Reads Per Kilobase of exon model per Million mapped reads (RPKM) values (A0 to M0), whereas there were differences between every unigene in terms of timing, which coincided with the phenomenon that ramie fiber has differing qualities during each harvest period [35]. Specifically, although the 13 unigenes were chosen because of their significant differences of expression in sample T compared with the other samples, the expression trends in SecS and ThiS were not as regular as those reflected by the RPKM values. Only isotig01514_77 and isotig06919_78 in SecS (Figure 2A2, B2) shared similar trends with the original (Figure 2A0, B0), while they showed dissimilar results in ThiS (Figure 2A3, B3). Meanwhile, three unigenes (isotig06943_25, isotig01251_6 and HRX1MBH01BLWDW_9) showed opposite expression patterns and two (isotig02054_18 and contig04902_19) had similar patterns in SecS and ThiS (Figure 2C2, C3; G2, G3; H2, H3 for the opposite trends and F2, F3; M2, M3 for similar patterns). The unigenes HRX1MBH01BLWDW_9 and isotig04660_8, isotig00610_25 and isotig09773_10 appeared to have opposite trends to the original (Figure 2H0, K0; I0, L0) in SecS (Figure 2H2, K2) or in ThiS (Figure 2I3, L3), respectively.

When we compared the 13 unigenes to investigate whether they were included in the two GO terms that were enriched in sample T (GO: 0030312 and GO: 0005576), six (isotig01251_6 and isotig02054_18 from the expansin family and isotig00610_25, isotig01663_22, isotig04660_8 and isotig09773_10 from the XTH family) were assigned to both GO terms, while two (isotig10345_23 from expansin and HRX1MBH01BLWDW_9 from the XTH families) were only included in the former GO term (GO: 0030312) and one (contig04902_19 from XTH family) was only assigned to the latter one (GO: 0005576). None of the specifically expressed cellulose synthase genes (isotig01514_77, isotig06919_78, isotig06943_25and isotig07154_19) were assigned to either of the two GO terms (Additional file 6).

### Additional features included in the transcriptome data

**Figure 4:**
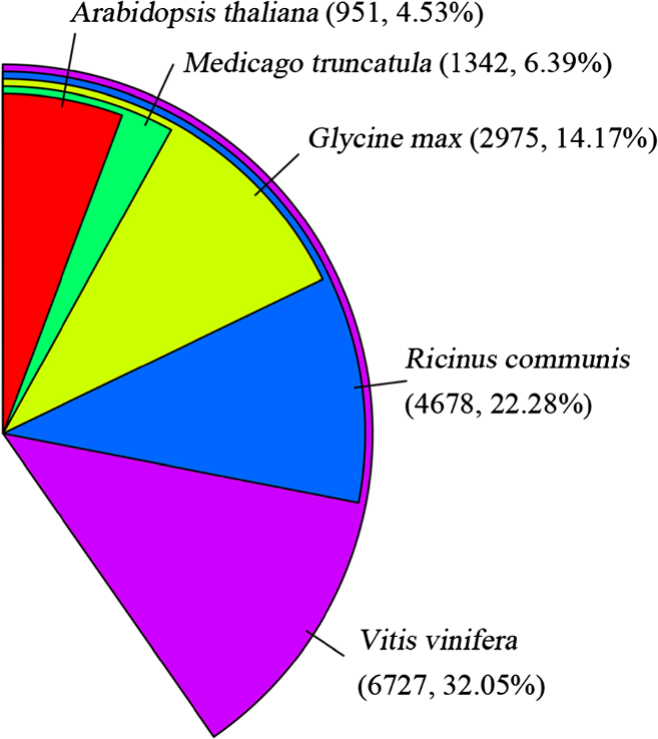
**Percentage numbers of the five most abundant annotated species.**

After annotation, the statistical numbers of sequences from different species that matched ramie unigenes were calculated from the annotation features. As displayed in Figure 4, the five most abundant species were *Vitis vinifera* (32.05%), *Ricinus communis* (22.28%), *Glycine max* (14.17%), *Medicago truncatula* (6.39%) and *Arabidopsis thaliana* (4.53%), representing around 80% of all the species that were annotated. This was very similar to previous results for ramie transcriptome sequencing [11]. Any dissimilarity may be attributed to the differences in the parts sampled in this study. In addition, the codon usage frequency was calculated (Additional file 11) for homologous sequence cloning using the COnsensus-DEgenerate Hybrid Oligonucleotide Primer (CODEHOP) method [36].

## Discussion

### Sample preparation and pyrosequencing

Ramie is a non-model highly heterozygosis perennial crop, known as “China grass”. Little previous genetic research has been conducted on this crop, especially on fiber development. To standardize experimental conditions, a fixed timeline was set in previous studies [11, 14]. However, the growth periods vary between ramie plants in different seasons; therefore, it is difficult for researchers to ascertain the exact standard (fixed timing or assured phenotypes) for sampling that represents the different fiber growth stages. In this study, we used the sampling method that has been successfully applied to flax [15, 16]. Accordingly, samples that represented no bast fiber cell formation (sample L), initial thickening (sample T), obvious thickening (sample M) and end of thickening (samples B) were generated (Figure 3A, B, C and D, respectively) from different parts of ramie from the start of fiber ripening (the aboveground stem about to turn pitchy, as indicated in Figure 3E). Correspondingly, the pyrosequencing output presented obvious and interesting dissimilarities among the four samples, represented as differentially expressed genes (Table 2 and Figure 1), and differentially enriched GO terms (Table 3) and KEGG pathways (Table 4). The 454 FLX + platform produced average read lengths in our library of 457 bp, which improved the subsequent *de novo* assembly results. However, the vague boundary of our sampling standard (difficult to ascertain the exact boundary among the four samples through different timings, as well as the initiation of the fiber ripening stage) resulted in the quantitative results in FirS (Figure 2A1 to M1) being dissimilar to the RPKM values (Figure 2A0 to M0), which suggested that our sampling method could be further standardized for future studies of ramie fiber development.

### The three gene families participating in ramie fiber development

Cellulose is the main component in plant cell walls [17]. Therefore, genes in the cellulose synthase superfamily, which have been shown to be associated with primary [37–39] or secondary [40–42] cell wall formation in *Arabidopsis*, were the first genes we investigated. A previous study identified 51 expressed sequence tags that belong to the cellulose synthase family [11] in ramie. Along with cellulose, polysaccharide xyloglucan is thought to play an important structural role in the primary cell walls of dicotyledons [26], which means that the XTH family, proven to have a function during the formation of secondary cell walls in vascular tissue [27] (especially in regulating cotton fiber elongation [28–30]), would be important in ramie fiber development. Finally, expansin, which has important roles in cell elongation [20] and expansion [21], is also associated with cotton fiber development [22, 24, 25]. Thus, we concentrated on the above three gene families in this study. As a result, most of the differentially expressed unigenes from these three gene families shared the all-up expression pattern in sample T (four out of five unigenes from the cellulose synthase superfamily, three out of four unigenes from the expansin gene family and six out of eight unigenes from the XTH gene family; Additional file 9), which showed that the early stage of ramie fiber growth (initiation of fiber cell thickening) is the most important for molecular regulation (especially for these three gene families) and for fiber development. In total, 96 unigenes showed the all-up expression pattern in sample T (Additional file 9), which should be subjected to further studies as a priority.

### Expression quantities of the unigenes that shared the all-up pattern in sample T

The variant numbers of all-up expressed unigenes (those that were respectively up-regulated in one of the four samples compared with the other three) in samples L (99) and T (96) compared with samples M (20) and B (13, listed in Additional file 9) were interesting. This result suggested that ramie grows more vigorously at the start of stem fiber growth. Furthermore, the results that: (1) two GO terms (GO: 0030312 and GO: 0005576) were concurrently enriched in sample T compared with the other three samples (samples L, M and B); and (2) most unigenes belonging to the three gene families (cellulose synthase superfamily, expansin gene family and XTH gene family) were all-up-regulated in sample T also suggested that the top part of the stem bark (sample T) was the most important part for fiber growth.

The results of qRT-PCR analysis of these 13 unigenes from similarly assayed samples across the whole growing season (FirS, SecS and ThiS) were interesting. First, similar trends between the FirS and RPKM results (A1 to M1 and A0 to M0, respectively) could indicate the effectiveness for the whole transcriptome data. Nevertheless, the quantitative result for isotig09773_10 was very different (Figure 2L1) to RPKM result (L0), and we could not determine the precise cause of this difference.

Second, despite the similar trends (expressed more abundantly in sample T than in samples M and B) for each unigene between FirS (Figure 2A1 to M1) and the RPKM results (A0 to M0), the relative expression quantities of each unigene in sample L were universally higher from reflected data (FirS) than that from the predicted data (RPKM). The sampling method was implicated as the origin of this discrepancy. As we previously mentioned, the stem shoot apex, including the top three (or so) young leaves, comprised sample L and contained no bast fiber (Figure 3A). Yet the upper part of the ramie stem (including sample L and sample T) grows more vigorously (more unigenes shared the all-up pattern in samples L and T), which made it more sensitive to environmental elements, such as temperature and sampling time. Additionally, despite the uniform sampling lengths and the validation of the slicing results (Figure 3), samples that were generated at different times (the samples for transcriptome sequencing and the corresponding quantitative experiments in FirS were generated in 2012 and in 2014, respectively) were still different.

Finally, despite the similarity in trends between the data from FirS (Figure 2A1 to M1) and RPKM (A0 to M0), which assured the reliability of the transcriptome results, discrepancies rose between the growth stages (FirS, SecS and ThiS). To determine their possible relationship with ramie fiber quality, further analysis was conducted. Among the 13 unigenes that were chosen for further quantitative experiments, six of them (isotig01251_6 and isotig02054_18 from the expansin family and isotig00610_25, isotig01663_22, isotig04660_8 and isotig09773_10 from the XTH family) were assigned to both GO terms (GO: 0030312 and GO: 0005576) that were enriched in sample T compared with the other three samples (samples L, M and B, displayed in Table 3). Furthermore, four of the six (isotig10345_23, isotig02054_18, isotig01663_22 and contig04902_19) unigenes shared congruent expression patterns in SecS and ThiS (Figure 2E2, E3; F2, F3; J2, J3 and M2, M3, respectively). If this phenomenon was relevant to the differences in ramie fiber quality during the three seasons, the slightly higher relevant expression quantities of sample T (compared with sample M or sample B) in SecS compared with ThiS (indicated by colors in Figure 2N) could further confirm this hypothesis. In support, *GhExp2*, which shared a high similarity with isotig02054_18 (based on the sequence BLAST results, data not shown), is expressed specifically in developing cotton fiber [24], while the sequence similarity was high between contig040902_19 and *AtXTH31*/ *AtXTH32*, which are highly expressed in tissues undergoing elongation/stem and shoot apical system [43]. Furthermore, considering the importance of xyloglucan-cellulose cross links in modulating the strength and extensibility of the primary plant cell wall, which is a key feature of classical models of this composite structure [44, 45] and that xyloglucan endo-transglycosylase activity, along with expansins, are the primary catalyst in cell wall loosening [43], we suggest that the early ramie fiber developmental stages were more sensitive to molecular regulation, which was reflected by the up-regulations of expansin and XTH genes (which control the deposition of cellulose fibrils, etc.). However, we could not draw any conclusions on whether these four unigenes were specifically relevant to ramie fiber fineness or crystallinity based on our limited results. Further studies should focus on the functional analysis or diversity among different cultivars during different seasons to improve our understanding of ramie fiber development. Finally, future studies should focus on identifying simple sequence repeats in unigenes of interesting sites and designing primers from them, to further our understanding of the underlying molecular systems.

### Additional features included in the transcriptome data

Although lacking of duplication in transcriptome data construction, the effectiveness of our transcriptome sequencing result was assured by a comparison of the quantitative output for FirS in 2014 (Figure 2A1 to M1) to RPKM values (Figure 2A0 to M0) conducted in 2012. Meanwhile, manual selection significantly improved the annotation information (Additional files 2, 3 and 4). In addition, the four most abundant species that were annotated to *Vitis vinifera*, *Ricinus communis*, *Glycine max* and *Medicago truncatula* (Figure 4) were similar to previous results (*Vitis vinifera*, *Ricinus communis*, *Populus trichocarpa* and *Glycine max*) [11], in which the authors declared that the Malpighiales (*Ricinus communis* and *Populus trichocarpa*), Fabales (*Glycine max*) and Rosales (ramie) are commonly placed in the superorder of rosids, while the relationship between Vitales (*Vitis vinifera*) and Rosales (ramie) is probably closer than that between Rosales, Malpighiales and Fabales [11]. In this study, our results further supported the relationship between Vitales (*Vitis vinifera*), Rosales (ramie), Malpighiales (*Ricinus communis*) and Fabales (*Glycine max* and *Medicago truncatula*). However, *Populus trichocarpa* did not appear in the most abundant annotated species list, which may indicate a dissimilarity of stem growth between ramie and *Populus trichocarpa*, especially in bast fiber development or cell wall thickening.

## Conclusions

In this study, transcriptome sequencing of samples from different ramie fiber developmental stages was carried out via pyrosequencing. The 58,369 unigenes (13,386 contigs and 44,983 isotigs) that were generated by *de novo* assembly had a high correlation with ramie fiber development, which increased our knowledge of the molecular mechanisms underlying bast fiber development in ramie. Most of the differentially expressed genes from the three targeted gene families (the cellulose synthase superfamily, the expansin gene family and the XTH gene family) shared an all-up expression pattern in sample T, which suggested that the early fiber developmental stage may play a crucial role in molecular regulation throughout the entire fiber growth period. These 13 unigenes, especially the four of them that from the expansin (two) and the XTH (two) families, could be applied in further in-depth expression and functional analyses. In addition, the sampling method used in this study could be applied and further standardized in other ramie fiber development studies.

## Methods

### Sample preparation and RNA extraction

Ramie cultivar 1504 (with a fiber finesse of 2800 m/g) was transplanted with pot from our Germplasm Resources Garden and was grown under natural conditions in 2010. Samples from the stem shoot (with about three leaves, sample L), the top part of stem bark (sample T), the middle part of stem bark (sample M) and the bottom part of stem bark (sample B, representing different stages of ramie fiber development, as displayed in Figure 3) were collected when the aboveground stem was turning pitchy in May 2012 (the first season of ramie fiber growth). Each sample was made up from three plants and consisted of samples that were about 5 cm long. They were mixed and frozen in liquid nitrogen for subsequent RNA extraction. Total RNA was separately extracted from the four samples, based on the method described previously [46]. Afterwards, the RNA quality was confirmed by gel electrophoresis and by NanoDrop 2000 spectrophotometer (Thermo, MA, USA).

### High throughput transcriptome sequencing

Transcriptome sequencing was performed on four equally pooled samples at Hanyu Biotech, Shanghai, China (http://www.hanyubio.com/) using the Roche 454 FLX + platform, according to the product manual. Briefly, after digestion by DNase I (Ambion, USA) at 37°C for 1 h, poly (A) RNA was isolated from 20 μg of total RNA using a Micropoly(A) Purist™ mRNA purification kit (Ambion, USA). Afterwards, cDNA was synthesized, based on a method described previously [47] with improvements: firstly, the first-strand cDNA was synthesized by Superscript II reverse transcriptase (Invitrogen, USA) at 42°C for 1 h using 10 μg of total RNA as template and GsuI-oligo dT as primer. Secondly, Dynal M280 magnetic beads (Invitrogen, USA) were used to select the mRNA/cDNA that ligated with biotin, which was performed by oxidizing the 5′ cap of mRNA using NaIO_4_ (Sigma, USA). After that, the first-strand cDNA was released by alkaline lysis, then added adaptor at the 5′ end by T4 DNA ligase (TAKARA, Japan) and synthesized the second-strand cDNA by Ex Taq polymerase (TAKARA, Japan). Finally, the polyA tail at 3′ end and the adaptor at 5′ end were removed by Gsu I. Before loading into the Roche 454 Genome Sequencer FLX + machine, the double-stranded cDNA with lengths ranging from 300 bp to 800 bp (broken by ultrasonoscope (Fisher, USA) and purified by Ampure beads (Agencourt, USA) immediately) were converted into a single strand template DNA (sstDNA) library using a GS DNA Library Preparation kit (Roche Applied Science, USA), and then fixed onto magnetic beads using GS emPCR kit (Roche Applied Science, USA). A whole run was performed from equivalent mixture of the four separately pooled samples. The sequence data generated in this study were deposited at the NCBI in the Short Read Archive (SRA) database under the accession number SRP040605.

### *De novo*assembling, annotation and GO terms/KEGG pathway construction

Raw reads, obtained from 454 pyrosequencing, were pre-processed by removing low quality reads (Q value <20) and assembled by Newbler under default parameters. The predicted protein coding sequences (obtained using the GetORF tool in EMBOSS [32]) were annotated to the non-redundant protein databases in GenBank and Swiss-Prot by BLASTp with a threshold of 1e-5. GO mapping was carried out using GoPipe software [33]. The predicted protein sequences were compared with the KEGG database [34] to obtain the KO numbers using bidirectional BLAST with threshold of 1e-5.

### Differential expression redundancy and enrichment analyses

After assembly and annotation, the universal reads from the four separately pooled samples were mapped to unigenes, and the RPKM values [48] were calculated. Then the differences between each unigene within the four samples were determined from the RPKM values using the MA-plot-based method with Random Sampling (MARS) model from the DEGseq program package [49], with a threshold of p-value less than 0.001. The differentially expressed genes were used in the GO terms/KEGG pathway enrichment analysis using the hyper geometric test to measure the significantly enriched terms. The formula was: . In this equation, *N* indicates the number of genes with GO/KO annotations and *n* represents the number of differentially expressed genes in *N*. The variables *M* and *m* represent the numbers of genes and differentially expressed genes, respectively, in each GO/KO term. The threshold used to determine the significant enrichment of a gene set was corrected to a P-value ≤0.05 and an FDR <0.01.

### Expression pattern analysis of the 13 unigenes in the counterpart samples during different seasons by qRT-PCR

Samples were analogously generated separately in the second (SecS) and third season (ThiS) in 2013 and in the first season (FirS) in 2014. Total RNA was extracted using the RNAprep Pure Plant Kit (Tiangen Biotech, Beijing, China) and reverse-transcribed by the GoScript Reverse Transcription System (Promega, WI, USA), following the product manual. Then qRT-PCR was performed on a Bio-Rad iQ5 Real-Time PCR System (Bio-Rad, CA, USA) using a final volume of 20 μl containing 1 μl cDNA sample, 10 μl iTaq Universal SYBR Green Supermix (Bio-Rad, CA, USA), 1 μl of each forward and reverse primers (10 μM each) and 7 μl nuclease-free water. The cycling parameters were 95°C for 5 min, followed by 40 cycles of 95°C for 15 s and 60°C for 30 s. Each sample was duplicated three times. Dissociation curve analysis was performed after each assay by ramping from 55°C to 95°C, at a rate of 0.5°C for 81 cycles and with each cycles held 6 s. Relative expression levels were calculated as described previously [50]. All primers for qRT-PCR (Additional file 12) were designed by the primer3 online tool (http://primer3.ut.ee/) and adjusted using oligo software version 7.56 [51]. *GAPDH* was selected as internal control based on preliminary experiments (data not shown).

## Authors’ information

Submitting author:

Jie Chen.

## Electronic supplementary material

Additional file 1: **Lengths distribution of separately pooled four samples as well as the overall aspect.** Read lengths of samples that separately pooled from stem shoot with leaves (sample L, A), top part of bark (sample T, B), middle part of bark (sample M, C), bottom part of stem bark (sample B, D) were displayed, as well as the overall read lengths (E) and lengths distributed from assembled contigs (F). (JPEG 527 KB)

Additional file 2: **Entries that obtained from BLAST results for further manual selection.** Fifteen entries of each unigene were obtained (if had that much) for manual selection. (XLSX 8 MB)

Additional file 3: **Unigenes with their annotation information altered after artificial selection.** Manual alteration was based on the tendency of 15 entries for each unigene that listed in Additional file 2. (XLSX 387 KB)

Additional file 4: **The final annotation results after artificial alteration.** (XLSX 4 MB)

Additional file 5: **GO distribution of assembled unigenes.** GO terms were distributed separately under cellular component, molecular function and biological processes. (DOCX 20 KB)

Additional file 6: **List of correspondence between GO terms and unigenes.** (TXT 8 MB)

Additional file 7: **Classification of KEGG pathways.** (DOCX 16 KB)

Additional file 8: **Detailed correlation of each unigene that attributed to KEGG pathways.** (XLSX 191 KB)

Additional file 9: **Detailed patterns of the unigenes that differentially expressed in four samples.** The patterns (up or down) were represented as the former one than the latter one in each combination. For instance, isotig00097_53 was down-regulated in sample T than sample B. (XLSX 59 KB)

Additional file 10: **Detailed expression quantities of each unigene.** (XLSX 10 MB)

Additional file 11: **Codon usages of each amino acid.** (XLSX 13 KB)

Additional file 12: **Primers for qRT-PCR quantification.** Primers were designed by primer 3 online tool (http://primer3.ut.ee/) and adjusted using Oligo software [51]. *GAPDH* was selected as internal control based on preliminary experiments (data not shown). Primers for *GAPDH* were carried out according to the sequence information (data not shown) that embodied in the transcriptome library. (XLSX 13 KB)

## Abbreviations

*CesA*: Cellulose synthase
CODEHOP: COnsensus-DEgenerate Hybrid Oligonucleotide Primer
*COMT*: Caffeic acid 3- O- methyltransferase
FDR: False discovery rate
FirS: the First Season
*GalAT*: α- 1,4- Galacturonosyltransferase
GO: Gene Ontology
KEGG: Kyoto Encyclopaedia of Genes and Genomes
KO: KEGG Orthology
qRT-PCR: Quantitative reverse transcribed PCR
RPKM: Reads Per Kilobase of exon model per Million mapped reads
SecS: the Second Season
ThiS: the Third Season
*UDPGDH*: UDP-glucose dehydrogenase
XTH: xyloglucan endotransglucosylase/hydrolase.
