# Supplementary figures and images for: Transcriptome profiling using pyrosequencing shows genes associated with bast fiber development in ramie (Boehmeria nivea L.)
Source: BMC Genomics. 2014 Oct 22;15(1):919. doi: 10.1186/1471-2164-15-919 (PMC4326285; doi:10.1186/1471-2164-15-919)

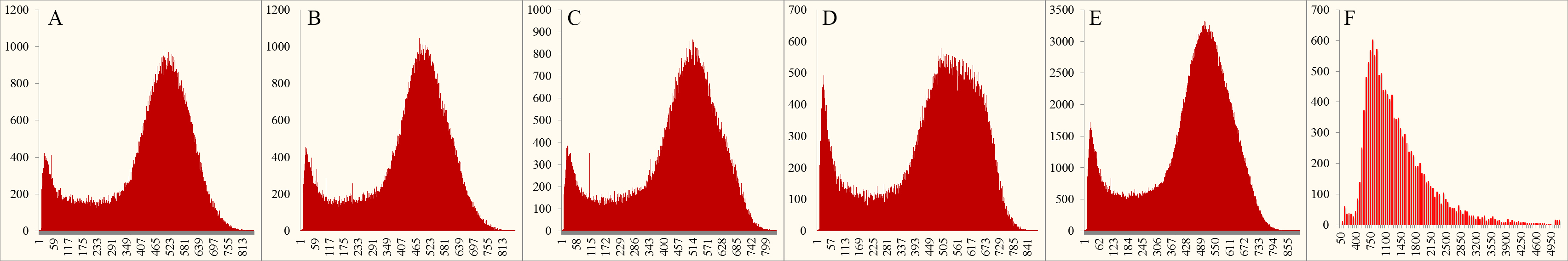

Supplement: Supplementary file 1 — Additional file 1: Lengths distribution of separately pooled four samples as well as the overall aspect. Read lengths of samples that separately pooled from stem shoot with leaves (sample L, A), top part of bark (sample T, B), middle part of bark (sample M, C), bottom part of stem bark (sample B, D) were displayed, as well as the overall read lengths (E) and lengths distributed from assembled contigs (F). (JPEG 527 KB) [file 12864_2014_6986_MOESM1_ESM.jpeg]
