# Supplementary material for: Transcriptome profiling using pyrosequencing shows genes associated with bast fiber development in ramie (Boehmeria nivea L.)
Source: BMC Genomics. 2014 Oct 22;15(1):919. doi: 10.1186/1471-2164-15-919 (PMC4326285; doi:10.1186/1471-2164-15-919)
Supplement: Supplementary file 2 [file 12864_2014_6986_MOESM5_ESM.docx]

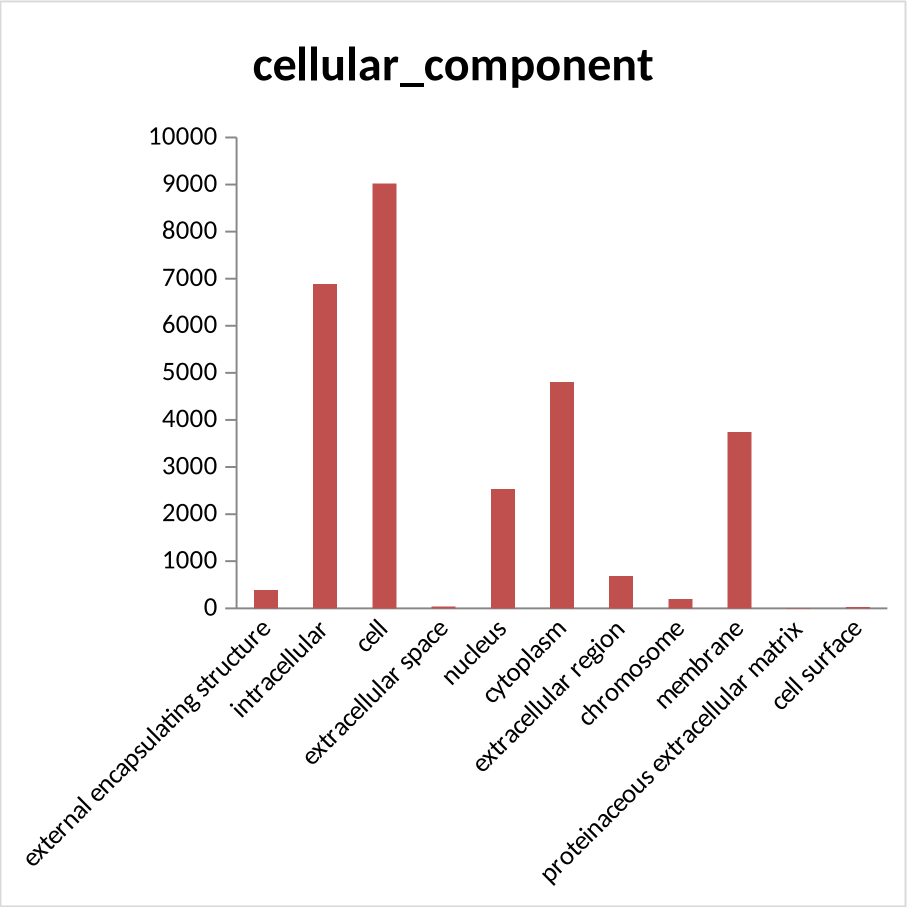
cellular_component
10000
9000
8000
7000
6000
5000
4000
3000
2000
1000
0
external encapsulating structure
intracellular
cell
extracellular space
nucleus
cytoplasm
extracellular region
chromosome
membrane
proteinaceous extracellular matrix
cell surface

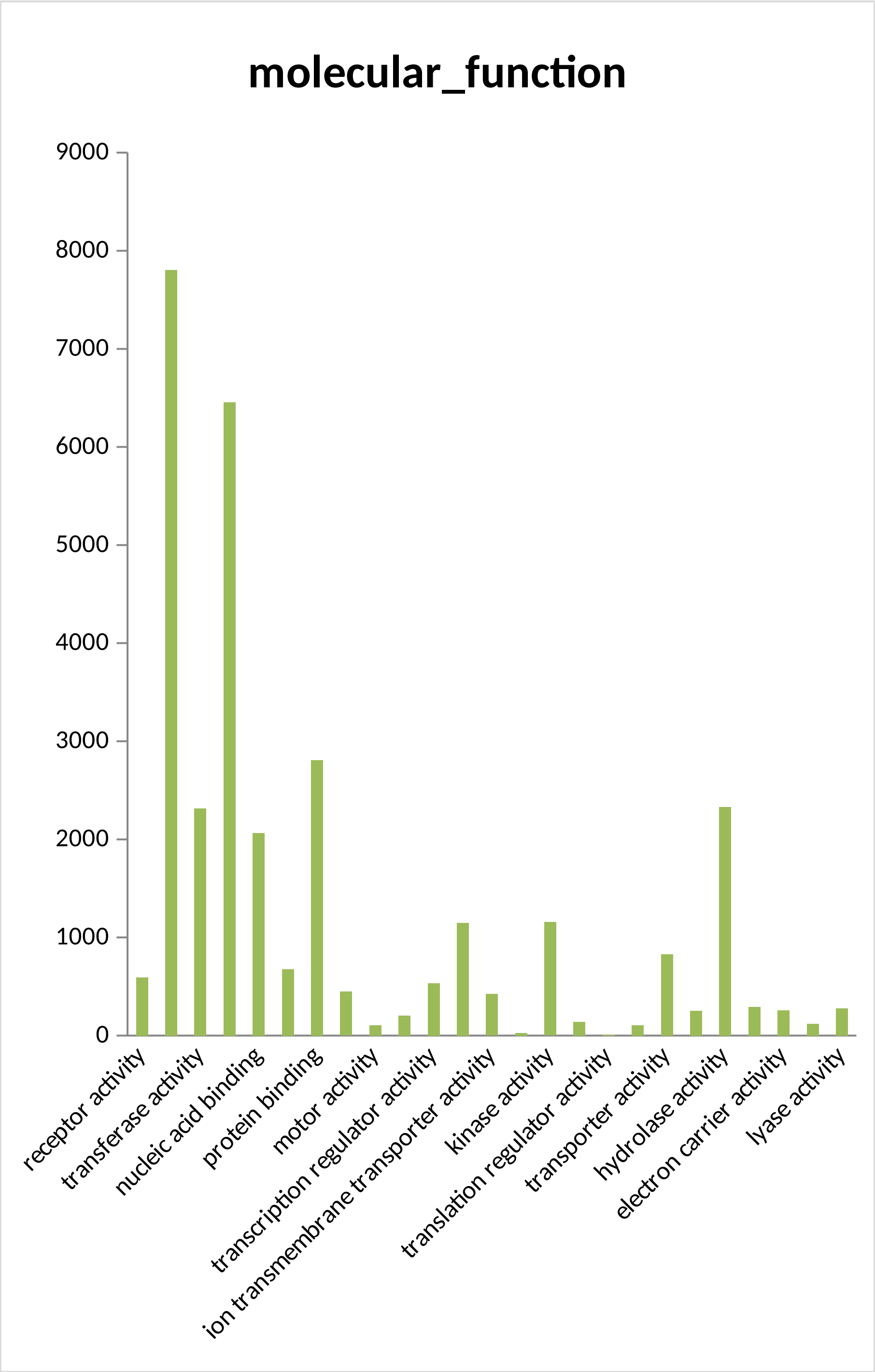
molecular_function
9000
8000
7000
6000
5000
4000
3000
2000
1000
0
receptor activity
transferase activity
nucleic acid binding
protein binding
motor activity
transcription regulator activity
ion transmembrane transporter activity
kinase activity
translation regulator activity
transporter activity
hydrolase activity
electron carrier activity
lyase activity

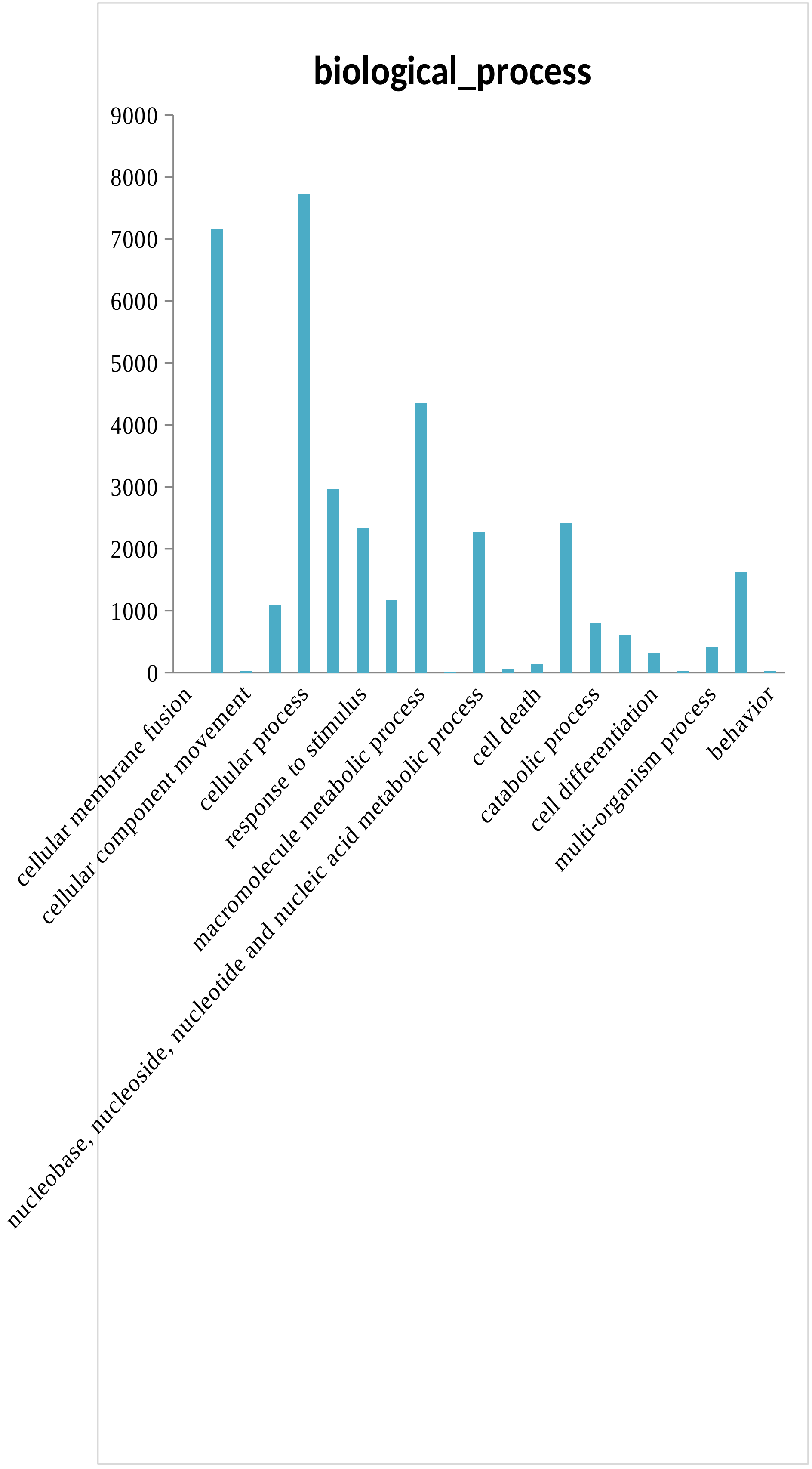
biological_process
9000
8000
7000
6000
5000
4000
3000
2000
1000
0
cellular membrane fusion
cellular component movement
cellular process
response to stimulus
macromolecule metabolic process
nucleobase, nucleoside, nucleotide and nucleic acid metabolic process
cell death
catabolic process
cell differentiation
multi-organism process
behavior
