# Supplementary material for: Transcriptome profiling using pyrosequencing shows genes associated with bast fiber development in ramie (Boehmeria nivea L.)
Source: BMC Genomics. 2014 Oct 22;15(1):919. doi: 10.1186/1471-2164-15-919 (PMC4326285; doi:10.1186/1471-2164-15-919)
Supplement: Supplementary file 3 [file 12864_2014_6986_MOESM7_ESM.docx]

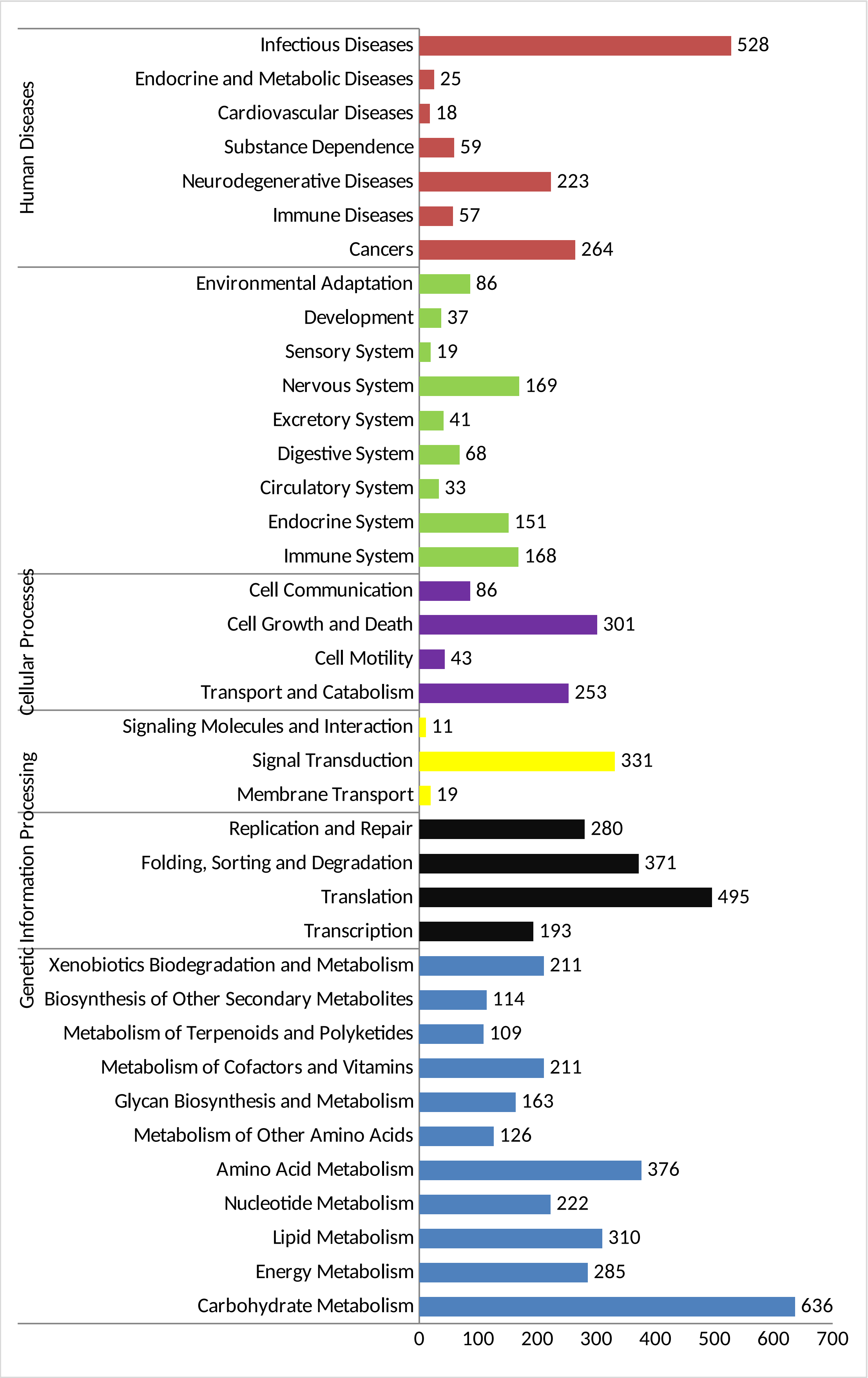
Human Diseases
Infectious Diseases 528
Endocrine and Metabolic Diseases 25
Cardiovascular Diseases 18
Substance Dependence 59
Neurodegenerative Diseases 223
Immune Diseases 57
Cancers 264
Environmental Adaptation 86
Development 37
Sensory System 19
Nervous System 169
Excretory System 41
Digestive System 68
Circulatory System 33
Endocrine System 151
Immune System 168
Cellular Processes
Cell Communication 86
Cell Growth and Death 301
Cell Motility 43
Transport and Catabolism 253
Signaling Molecules and Interaction 11
Signal Transduction 331
Membrane Transport 19
Genetic Information Processing
Replication and Repair 280
Folding, Sorting and Degradation 371
Translation 495
Transcription 193
Xenobiotics Biodegradation and Metabolism 211
Biosynthesis of Other Secondary Metabolites 114
Metabolism of Terpenoids and Polyketides 109
Metabolism of Cofactors and Vitamins 211
Glycan Biosynthesis and Metabolism 163
Metabolism of Other Amino Acids 126
Amino Acid Metabolism 376
Nucleotide Metabolism 222
Lipid Metabolism 310
Energy Metabolism 285
Carbohydrate Metabolism 636
0 100 200 300 400 500 600 700
